# Comprehensive Catalog of Variants Potentially Associated with Hidradenitis Suppurativa, Including Newly Identified Variants from a Cohort of 100 Patients

**DOI:** 10.3390/ijms251910374

**Published:** 2024-09-26

**Authors:** Kévin Muret, Vincent Le Goff, Claire Dandine-Roulland, Claire Hotz, Francette Jean-Louis, Bertrand Boisson, Lilia Mesrob, Florian Sandron, Delphine Daian, Robert Olaso, Edith Le Floch, Vincent Meyer, Pierre Wolkenstein, Jean-Laurent Casanova, Yves Lévy, Eric Bonnet, Jean-François Deleuze, Sophie Hüe

**Affiliations:** 1 Centre National de Recherche en Génomique Humaine (CNRGH), Institut de Biologie François Jacob, CEA, Université Paris-Saclay, 91000 Evry, France; kevin.muret@cnrgh.fr (K.M.);; 2 Public Health Department, Henri-Mondor Hospital, Assistance Publique-Hôpitaux de Paris (AP-HP), 94000 Créteil, France; 3 Transversal Dermatology Unit, Jacques Puel Hospital Center, 12000 Rodez, France; 4 Team 16, Vaccine Research Institute (VRI), INSERM U955, Institut Mondor de Recherche Biomédicale (IMRB), Henri-Mondor Hospital, UPEC, 94000 Créteil, France; 5 St. Giles Laboratory of Human Genetics of Infectious Diseases, Rockefeller Branch, The Rockefeller University, New York, NY 10065, USA; 6 INSERM U1266, Institute of Psychiatry and Neuroscience of Paris (IPNP), Paris Cité University, 75014 Paris, France; 7 Imagine Institute, Paris Cité University, 75015 Paris, France; 8 Pediatric Hematology-Immunology and Rheumatology Unit, Necker Hospital for Sick Children, Assistance Publique-Hôpitaux de Paris (AP-HP), 75015 Paris, France; 9 Laboratory of Human Genetics of Infectious Diseases, Necker Branch, INSERM U1163, Necker Hospital for Sick Children, 75015 Paris, France; 10 Howard Hughes Medical Institute, New York, NY 10032, USA; 11 Centre d’Etude du Polymorphisme Humain (CEPH), Fondation Jean Dausset, 75010 Paris, France; 12 Centre de Référence, d’Innovation, d’Expertise et de Transfert (CREFIX), 91000 Evry, France; 13 Biologic Immunology-Hematology Department, DMU Biologie, Henri-Mondor Hospital, Assistance Publique-Hôpitaux de Paris (AP-HP), 94000 Créteil, France

**Keywords:** hidradenitis suppurativa, whole-exome sequencing, γ-secretase, nicastrin, notch pathway, inflammation

## Abstract

Hidradenitis suppurativa (HS) is a chronic skin disease characterized by painful, recurrent abscesses, nodules, and scarring, primarily in skin folds. The exact causes of HS are multifactorial, involving genetic, hormonal, and environmental factors. It is associated with systemic diseases such as metabolic syndrome and inflammatory bowel disease. Genetic studies have identified mutations in the γ-secretase complex that affect Notch signaling pathways critical for skin cell regulation. Despite its high heritability, most reported HS cases do not follow a simple genetic pattern. In this article, we performed whole-exome sequencing (WES) on a cohort of 100 individuals with HS, and we provide a comprehensive review of the variants known to be described or associated with HS. 91 variants were associated with the γ-secretase complex, and 78 variants were associated with other genes involved in the Notch pathway, keratinization, or immune response. Through this new genetic analysis, we have added ten new variants to the existing catalogs. All variants are available in a .vcf file and are provided as a resource for future studies.

## 1. Introduction

Hidradenitis suppurativa (HS) is a debilitating dermatological disorder that affects approximately 1% of the global population. It is characterized by the formation of large suppurative abscesses, sinuses, nodules, and scars in intertriginous areas, including the axillae, groin, and/or anogenital regions [1]. Although the pathogenesis of HS is multifactorial and poorly understood, its pathomechanism is very likely linked to aberrant keratinization and autoinflammation. Consequently, HS is recognized as an autoinflammatory keratinization disease [2]. It remains uncertain whether autoinflammatory events precede or follow the hyperkeratotic changes in the hair follicle epithelia, although follicular occlusion is typically considered the primary event [3]. Several factors, including genetics, microbiota, and environmental factors such as obesity and smoking, may act as triggers or increase the risk of suffering from HS. Many patients also suffer from syndromic forms of HS or have comorbidities [4,5,6,7,8], such as inflammatory bowel disease (IBD), with examples including ulcerative colitis or Crohn’s disease (Supplementary Figure S1).

Only a small proportion of patients with familial or syndromic HS are monogenic (5%), despite the estimated high heritability of HS (77–80%) [9]. Mutations in genes encoding γ-secretase, an intramembrane multisubunit protease complex, have been identified in several members of Chinese families with severe HS [10]. The γ-secretase complex comprises four protein subunits: the anterior pharynx-defective protein APH-1A or APH-1B (*APH1A/B* genes), the nicastrin protein NCSTN (*NCSTN* gene), the presenilin protein PS-1 or PS-2 (*PSEN1/2* genes), and the presenilin enhancer PEN-2 (*PSENEN* gene). It has been established that γ-secretase proteolyzes the transmembrane domain of more than 100 substrates, including those derived from the amyloid precursor protein (APP) and the Notch family of cell surface receptors [11]. To date, 91 mutations have been described in the γ-secretase complex, with more than half located in the nicastrin subunit. Despite several articles implicating γ-secretase variants in HS, evidence for direct causal mechanisms is lacking [12]. Given the pivotal functions of Notch in maintaining epidermal and follicular homeostasis as well as regulating inflammatory processes, Notch deregulation has been proposed to underlie the molecular basis of HS observed in patients with pathogenic variants in γ-secretase protein-coding genes [13].

Furthermore, many other genes have been identified as potentially implicated in HS: *AIM2* [14], *DCD* [15], *DEFB126* [16], *FGFR2* [17,18], *GJB2* [19,20,21,22,23,24], *IL1RN* [25], *IRF2BP2* [26], *KDF1* [27,28], *KRT6A* [29], *KRT17* [30,31], *MEFV* [17,24,25,32,33,34], *NBPF12* [16], *NF1P6* [16], *NLRC4* [24], *NLRP3* [25], *NOD2* [17,24,25,33], *NOTCH1* [35], *NOTCH3* [16,36], *NOTCH2NLA* [16], *OCRL* [37], *OTULIN* [24], *POFUT1* [38,39], *POGLUT1* [40], *PSTPIP1* [17,24,25,32,36,41,42,43,44,45,46,47,48], *PSMB8* [25], *RORC* [16], *SLC46A2* [16], *TCIRG1* [16], and *WDR1* [24]. Variants in these genes are often found in individuals with syndromic forms of HS. However, the precise contribution of each gene in HS development is difficult to determine.

Although no fully penetrant variants causing multigenerational disease have been identified, the high heritability rates suggest that sporadic forms of HS have a significant genetic component contributing to their etiology [49]. However, the precise nature of the genetic variants causing common non-syndromic forms of HS is still unclear. Here, we performed whole-exome sequencing (WES) on a cohort of 100 individuals with HS and used a control cohort of 100 individuals without HS from the French Exome consortium [50,51]. We examined genetic variants in genes described in the literature as being associated with the disease. We identified seven variants in the *NCSTN* gene, three of which were not described in dbSNP but were found only in articles. We pinpointed eight other variants (including two new ones) in the other γ-secretase complex genes. Finally, we found 21 variants in our HS individuals that have been described in the literature affecting other genes than γ-secretase genes, of which only three are of real interest (absent in our controls and with a high deleteriousness score). Moreover, we added two noteworthy new variants in the *SLC46A2* and *NOTCH3* genes.

## 2. Results

### 2.1. Description of the Cohort

We collected data from 100 patients with HS and gathered detailed clinical characteristics (Table 1). Age at enrollment was 34.7 ± 11.9 years; 64 were female (sex ratio 2:1). Twenty-four were obese (BMI ≥ 30), and more than 50% were smokers. Four families were included, and 28 patients reported having an affected first-degree relative. Hurley phenotypes, as described by van der Zee and Jemec [52], were categorized at enrollment, with the majority exhibiting the regular phenotype.

### 2.2. Variants Identified in Nicastrin (NCSTN) Gene

**Figure 1:**
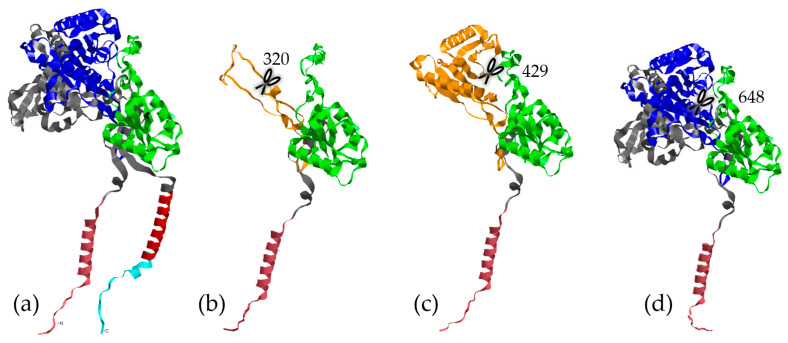
Computational models of the NCSTN protein. In cyan, red, and grey: the cytosolic, transmembrane (TM), and extracellular regions or domains. In pink: the signal peptide responsible for addressing the protein to the membrane. In green: the small lobe that would interact with the substrate. In dark blue: the large lobe. (**a**) Reference (WT), (**b**) p.Ala300TrpfsTer20, (**c**) p.Arg429*, and (**d**) p.Trp648* variant models.

As most known variants identified in HS patients are located in NCSTN (~37%) (Figure 1a), we started analyzing SNVs in the *NCSTN* gene. We identified seven variants, three of which have not been described in dbSNP or gnomAD, and whose bioinformatic predictions indicate that they might have a major impact on the protein structure.

The first variant, p.Leu17SerfsTer30 (c.47dupG; GRCh38:1: 160343443), is a frameshift variant that truncates the protein very prematurely (Supplementary Figure S2) and is expected to induce a nonsense-mediated decay (NMD) reaction to eliminate the aberrant transcript. This heterozygous variant is present in a 43-year-old woman with a family history of HS.

The second variant, p.Ala300TrpfsTer20 (c.896_897dupTG; GRCh38:1:160352104), is another frameshift variant that truncates the protein at position 320 (Figure 1b). This would result in a protein without its active site (in orange) and its transmembrane domain (in red), which prevents it from integrating into the cell membrane. It would therefore produce a non-functional protein or a γ-secretase haploinsufficiency, even though the mutation is heterozygous. Although PhyloP shows a score of −7.97, demonstrating rapid evolution of this site in mammals, this specific variant has a CADD Phred-like score of 34 (i.e., in the 0.04% most deleterious variants). It affects three HS individuals in the same family: a father (I:1) and his two daughters (II:1 and II:2).

The third and fourth variants are nonsense variants p.Arg429* (Figure 1c) (rs771414318 already described in a Japanese individual [53]) and p.Trp648* (Figure 1d) (not known in dbSNP or gnomAD), truncating the protein beyond the small lobe of the nicastrin. The transmembrane domain, such as the two previous variants, would be lost. These variants are found in sporadic cases of HS affecting a 50-year-old man and a 39-year-old woman, respectively. As expected, their CADD Phred-like scores are very high (39 and 51, respectively), and these variants are in fairly conserved regions (PhyloP = 7.04 and 4.42). It is important to note that the p.Trp648* variant has a SpliceAI score that is very close to 0.5, suggesting a potential gain of a splice acceptor site.

Additionally, we identified three lower-impact variants: two missense p.Glu77Asp (c.231G>C) and p.Asn417Tyr (c.1249A>T), and an intronic variant c.1180-5C>G, all of which have been described in the literature [17,36,54]. The p.Glu77Asp variant (rs35603924) (Supplementary Figure S2) is found in a 17-year-old woman with a Hurley stage III HS. Although very rare in the European population, this variant is not so rare in the African population (AF~6%) and does not seem, according to prediction tools, to have a strong impact on the protein. Unfortunately, we do not have information about the phototype of this young woman. The second missense variant, p.Asn417Tyr (rs143039637), affects a 33-year-old woman who also has Hurley stage III HS. This is a non-familial case. This variant is very rare and, according to SIFT and PolyPhen-2, appears to be “deleterious” and “possibly damaging”. Finally, the intronic variant c.1180-5C>G (rs7528638), although described in the literature [54], is common in the population with a frequency of over 5%. Similarly, in our cohort, 7.07% of patients and 12% of controls carry this variant. The SpliceAI and Pangolin prediction tools have scores below 0.5, which does not indicate a high probability of alternative splicing induced by this variant.

### 2.3. Variants Identified in Other Genes Involved in the γ-Secretase Complex

Approximately 20% of variants associated with HS and described in the literature affect other γ-secretase genes other than nicastrin. Yet we report four variants for PS-1, one for PS-2, and three for APH-1B (Supplementary Figure S2), of which three, one, and two variants, respectively, are known from dbSNP and gnomAD.

The most interesting variant is a c.554del(A) frameshift deletion (p.Lys185SerfsTer10, rs745918508), which causes a truncation of the last third of the APH-1B protein. It is observed in a 36-year-old woman with no family history. Although it remains very rare in the population (0.007%), it was also found in one of our control individuals. The CADD Phred-like score of 27.8 is very close to the Ensembl deleteriousness threshold of 30.

The two other variants in the *APH1B* gene are missense SNVs. The p.Thr27Ala (rs77834210) variant affects two unrelated individuals, one of whom is a phototype 6 male, which is consistent with the greater presence of this variant in the African population (1.05% vs. 0.33% in non-Finnish Europeans). The other p.Leu71Val variant affects only one woman. This second variant has a very high probability of being deleterious (SIFT = 0.01; PolyPhen-2 = 0.998).

Of the four variants detected in the *PSEN1* gene, we report a new one: p.Gln325Glu in a single individual, which probably has no particular effect (only the REVEL score exceeds its threshold of 0.5). The other three variants have already been described in the literature [36,54,55] but do not have a strong impact on protein structure. These are the variants c.868+16G>T (rs165932), p.Glu318Gly (rs17125721), and c.1248+8T>C (rs362382). The first two are shared by more controls than patients in our cohort (82 vs. 80% and 5 vs. 4%), which is perfectly consistent with what is known from gnomAD: 58% and 1% of the world population. The last variant showed scores of 0 for SpliceAI and Pangolin, demonstrating a null probability of splice site alteration.

Finally, we detected a new variant in the *PSEN2 gene*: p.Gly34Ser, which is not listed in the literature or databases and is only carried by an individual with no family history. According to the prediction tools, this variant would not be considered deleterious.

### 2.4. Comprehensive Catalog of γ-Secretase Variants

To provide a useful resource for the medical and scientific communities, we have listed all the variants affecting γ-secretase described in the literature in patients with HS with or without comorbidities. They are listed in Table 2 and Supplementary Table S1. To ensure the resource is of high quality and value, we have provided the positions of the variants on different reference genomes in relation to the reference transcript defined by the MANE project, the aim of which is to harmonize the annotations of genes and transcripts. We have identified numerous errors in recent reviews (e.g., Supplementary Table S2) and have corrected them in this article. The systematic verification of all variants requires a certain level of expertise and is very time-consuming when the variants are poorly annotated in articles. To avoid future issues, we also provide the left-normalized .vcf file of all variants discussed in this article as Supplementary Data S1 to aid future studies and reviews.

### 2.5. Variants Identified in Other Genes Described as Being Associated with HS

To present an exhaustive review of all known variants described in the literature with a potential impact or link to HS, we also analyzed the 78 known SNVs that are not related to γ-secretase. These genes include *AIM2* [14], *DCD* [15], *DEFB126* [16], *FGFR2* [17,18], *GJB2* [19,20,21,22,23,24], *IL1RN* [25], *IRF2BP2* [26], *KDF1* [27,28], *KRT6A* [29], *KRT17* [30,31], *MEFV* [17,24,25,32,33,34], *NBPF12* [16], *NF1P6* [16], *NLRC4* [24], *NLRP3* [25], *NOD2* [17,24,25,33], *NOTCH1* [35], *NOTCH3* [16,36], *NOTCH2NLA* [16], *OCRL* [37], *OTULIN* [24], *POFUT1* [38,39], *POGLUT1* [40], *PSTPIP1* [17,24,25,32,36,41,42,43,44,45,46,47,48], *PSMB8* [25], *RORC* [16], *SLC46A2* [16], *TCIRG1* [16], and *WDR1* [24]. They are primarily involved in the Notch signaling pathway, immune response pathway (principally the inflammasome), and keratinization, as described by Jfri et al. [118] (Supplementary Figure S3). Note that we cannot compare the proportions of variants between those in the γ-secretase complex and those in other genes, since many studies have only performed targeted exome sequencing and have not examined all of these genes.

We found 21 variants in our cohort in common with published studies (Supplementary Tables S3 and S1). Of these, eight variants had an allelic frequency > 2%, and five variants had a frequency between 1 and 2%. Among the remaining eight variants, five missense variants had no scores indicating deleteriousness, while the last three variants were of particular interest: *RORC*:p.Arg10* (rs17582155) [16], *GJB2*:p.Glu114Gly (rs2274083) [19], and *NOD2*:p.Ala891Asp (rs104895452) [17]. Their allelic frequencies are 0.279, 0.044, and 0.545% in the general population, respectively.

The RORC p.Arg10* variant is a stop codon gain that truncates the protein from the 10th amino acid (CADD Phred-like score = 36). This variant affects two unrelated HS individuals in our cohort, including the youngest daughter (II:2) in the family with the NCSTN p.A300Wfs*20 mutation. The father (I:1) and the other daughter (II:1) (Hurley II) do not carry this RORC variant, whereas the youngest daughter (II:2) has a more severe form of the disease (Hurley III). The *GJB2* variant is a missense mutation with a CADD Phred-like score of 20.8, affecting a sporadic case of HS. The *NOD2* variant is a missense mutation with a CADD Phred-like score of 25.5 and the most deleterious SIFT and PolyPhen-2 scores (0 and 1, respectively). Complementary analyses using missense3D and AlphaMissense describe this mutation as having a “neutral” and “likely benign” effect. This variant affects two unrelated individuals.

Additionally, we highlight two other high-impact variants among the 29 genes reported in the literature that do not affect the individuals in our control cohort. These are two frameshift variants: one affecting *SLC46A2* (rs1841700210-p.Ala326GlyfsTer133) and the other affecting *NOTCH3* (rs749829137-p.Cys43LeufsTer32). As expected, their CADD Phred-like scores are close to 30.

## 3. Discussion

We analyzed WES data from 100 HS patients, predominantly non-syndromic cases, with a focus on genes already reported to be mutated in HS. We identified 15 variants in the γ-secretase complex, including 12 with rare frequencies (<1%) in the general population. These 12 variants were found in 15 patients, including a family of three individuals affected by the *NCSTN* p.A300Wfs*20 variant. Thus, 15% of the sporadic cases in our HS cohort have a variant in the γ-secretase complex with a predicted moderate to strong effect.

A detailed literature review allowed us to compile a catalogue of potentially HS-causing variants in the γ-secretase complex. Table 2 lists the 91 already known variants and eight new variants discovered in our HS1 and HS2 cohorts, over two-thirds of which are predicted to have a significant impact on γ-secretase function (e.g., nonsense mutations, frameshifts leading to premature truncation, alternative splicing, or elongation). Approximately 40% of these variants are documented in gnomAD (v. 4.1-exome), an additional 11% are known in dbSNP, and the remaining 49% are only described in articles. Among these, only four variants have an alternative allele frequency greater than 1% in the general population and non-Finnish Europeans: c.436+129A>G (*NCSTN*), c.1180-5C>G (*NCSTN*), c.868+16G>T (*PSEN1*), and c.953A>G (*PSEN1*; p.E318G), with frequencies of 3.6, 5.1, 57.6, and 1.8%, respectively. These variants have been previously reported [42,54,55] with noted frequencies in the general population or in intrafamilial non-HS controls. Functional studies of some of these variants, such as p.Val75Ile [29], p.Asp185Asn [30], p.Pro211Arg [31], and p.Gln216Pro [29], have shown no impact on γ-secretase activity, suggesting that they are unlikely to be implicated in HS. These findings underscore the necessity for functional studies to elucidate the functional impact of these variants.

Despite its relatively small size of 101 amino acids, the PEN-2 protein has 20 identified variants in the PSENEN gene, with 95% of these having a strong effect. The literature describes that the four proteins of the γ-secretase complex are crucial for its proper function, and deregulation of any single protein can destabilize the entire complex [119]. For instance, PEN-2 is necessary for PS1 endoproteolysis [120], and PS1 mediates NCSTN maturation and intracellular trafficking [119]. Therefore, a strong-effect variant in one of the γ-secretase subunits is likely to have severe functional consequences. It has been demonstrated that knockdown of NCSTN in HaCaT cells disrupts the interaction between PEN-2 and PS1, impairing γ-secretase activity and leading to abnormal keratinocyte differentiation [64].

In the article by de Oliveira et al. [96], the authors demonstrate that the heterozygous nonsense mutation c.131T>A (p.L44*) in the *NCSTN* gene activates the NMD mechanism, which degrades the aberrant transcript. This degradation can be reversed by the addition of gentamicin, restoring the expression of the truncated NCSTN protein. The resulting haploinsufficiency can affect the expression of genes related to the type I interferon response pathway [121]. Moreover, based on the pLI, pRec, and pNull scores (respectively the probabilities of intolerance to loss of function, being a recessive gene, or being an unconstrained gene) associated with the genes described in this article, *NCSTN* is among the genes most prone to haploinsufficiency (Supplementary Figure S4). However, Pink et al. [86] still assert that haploinsufficiency alone is not sufficient to cause HS.

Among the γ-secretase complex variants reported in this article, there are five heterozygous nonsense or frameshift variants that could potentially lead to the degradation of *APH1B* and *NCSTN* RNA. Therefore, these variants warrant further functional analysis to assess their potential role in HS.

Finally, variants affecting proteins other than those of the γ-secretase complex primarily concern syndromic cases of HS or cases with co-morbidities (as noted in Supplementary Figure S1). Variants in GJB2 are more commonly associated with the Follicular Occlusion Triad (FO3) and Keratitis-Ichthyosis-Deafness syndrome (KID), KRT17 variants with the Follicular Occlusion Tetrad (FO4), KRT6A and KRT17 variants with Pachyonychia Congenita (PC), MEFV variants with PASH and PAPASH syndromes, OCRL variants with Dent Disease 2 (DD2), POFUT1 and POGLUT1 variants with Dowling Degos Disease (DDD), and PSTPIP1 variants with PASH and PAPASH syndromes. In these cases, it is even more challenging to establish the causative role of the variants in HS. For example, the p.D50N mutation is associated with HS patients who also have KID syndrome [19]. This variant is also found in KID patients without HS, underscoring the importance of careful interpretation of variant data. The two variants that we include in this catalog concern the *NOTCH3* and *SLC46A2* genes. The first gene is directly involved in the Notch signaling pathway; the second gene is involved in the activation of NOD (nucleotide oligomerization domain) receptors in epithelial cells and initiates an anti-inflammatory response [122]. Both genes are highly deregulated in the lesional tissues of HS patients [123,124]. Finally, it is also important to note that this catalog complements existing data. While recent studies suggest that known variants in *NOD2* are likely risk polymorphisms with low effect sizes [125], this does not account for the variant identified by de Oliveira et al. in 2022 [17] (rs104895452), which we include here, and which has scores indicating a high probability of being deleterious.

This review remains limited by the lack of uniformity in the methods used to study variants across different articles. Only 23.46% and 1.68% of the variants were identified through WES and WGS, respectively, while over half were identified by targeted sequencing methods, including Sanger sequencing of a gene segment, single gene sequencing, γ-secretase gene panels, or broader gene panels. Additionally, 10.06% of the variants were detected through RNA-seq data, and the remaining variants were identified through association studies or burden tests. Importantly, 24.02% of the variants in the catalog were validated by two different methods from two independent studies, and 2.79% were validated by three methods. The criteria applied to variant selection, filtering, and validation were specific to each study, making it evident that the heterogeneity of these analyses prevents the creation of an exhaustive catalog of variants for each case.

In conclusion, our study has identified and cataloged 10 new and 91 and 78 previously known variants in the γ-secretase complex and other genes, respectively, potentially associated with HS. These findings highlight the complex genetic landscape of HS, proposing both common and rare variants that may contribute to its pathogenesis. Our analysis emphasizes the potential role of rare variants in the disease’s development and suggests that both haploinsufficiency and more complex genetic interactions are maybe involved. Notably, the presence of multiple low-impact common variants may create a genetic predisposition that, in combination with environmental factors and lifestyle conditions (e.g., smoking behavior or obesity), facilitates the onset of the disease. Further functional studies are necessary to elucidate the precise mechanisms by which these genetic alterations influence HS. Understanding these mechanisms may provide new insights into targeted therapeutic strategies, offering hope for more effective treatments for individuals affected by this debilitating condition.

## 4. Materials and Methods

### 4.1. Sample Collection

This article combines data from three independent cohorts: the HS1 cohort, which includes 75 PBMC samples from HS patients; the HS2 cohort, comprising 25 diverse samples (PBMC, keratinocytes, ORS cells in culture, as well as total dermal cells); and the CTL cohort, consisting of 100 non-HS control individuals from the public French Exome consortium [50,51] (http://lysine.univ-brest.fr/FrExAC/, accessed on 10 September 2022). We maintained exactly the same male-to-female ratio between the HS and CTL cohorts. The characteristics of the 100 HS patients are summarized in Table 1. The ethics committee of Henri Mondor Hospital (CPP n°10-026), in agreement with the Declaration of Helsinki, approved this study, and written informed consent was received from participants before inclusion in this study. All patient data for the HS1 and HS2 cohorts are available in Supplementary Table S4.

### 4.2. Whole Exome Sequencing

Whole Exome sequencing was performed using the Illumina HiSeq2500 sequencer for the HS1 cohort, the HiSeq4000 for the HS2 cohort, and the HiSeq2000 and HiSeq2500 (Illumina, Inc., San Diego, CA, USA) for the CTL cohort according to the following kit specifications (Table 3).

In order to solely focus on informative variants across the different cohorts, only the variants shared between the kits were analyzed. This mainly excludes UTR regions and approximately 4% of CDS coverage (based on the oldest kit). No batch effect was detected despite the use of different protocols.

### 4.3. Variant Calling and Annotation

The individual .g.vcf files were obtained through an in-house pipeline using the following tools: bwa-mem (v. 2.2.1) [126,127], PicardTools (v. 2.26.9) [128], Samtools (v. 1.16) [129], Sambamba (v. 0.8.1) [130], GATK (v. 4.2.3.0) [131,132], and bedtools (v. 2.30.0) [133]. The alignment was performed on the reference genome, GRCh38. The .g.vcf files were aggregated and then transformed into .vcf files using the GATK HaplotypeCaller and GenotypeGVCFs modules. The variants were then limited to the regions captured by the kits (extended from 10 to 100 bp) and filtered according to the commonly used criteria [134]: (1) for SNVs: QD < 2, QUAL < 30, SOR > 3, FS > 60, MQ < 40, MQRankSum < −12.5, and ReadPosRankSum < −8; (2) for Indels: QD < 2, QUAL < 30, FS > 200, and ReadPosRankSum < −20. We annotated our variants with the gnomAD (v. 4.1-exome) and dbSNP 2.0 (v. 152) [135] databases using the SnpEff tool [136] and its SnpSift module (v. 4.5) [137]. We complemented missing data with the online tool CADD (v. 1.7) [138]. Variant allele frequencies and deleteriousness scores (using various tools) are provided by gnomAD (v. 4.1). The thresholds used to assess the deleteriousness of a variant according to the tools are as follows:

- CADD Phred-like score ≥ 30: among the 0.1% most deleterious variants;
- REVEL ≥ 0.5: deleterious missense;
- SIFT < 0.05: deleterious missense;
- AlphaMissense > 0.564: possibly deleterious;
- PolyPhen-2 ≥ 0.85: probably damaging missense;
- SpliceAI ≥ 0.8: variant with the probability of altering splicing;
- Pangolin ≥ 0.8: variant with the probability of altering splicing;
- PhyloP (mammals) < 1.5: fast evolution of the locus in mammals.

### 4.4. Variant Validation

Each variant of the literature was validated manually. The data retrieved from the articles (electrophoregrams, sequences, genome used, HGVS, dbSNP identifier, transcript concerned, gene position, etc.) were compared with the Ensembl and/or NCBI databases and MANE reference transcripts to harmonize the data. Where data was missing, it was complemented (e.g., if position or exon information was absent, these details were incorporated into the final tables). The tools and databases used were mainly LitVar^2^ (v. 2.0) [139], MutationTaster (v. 2021) [140], gnomAD (v. 2.1.1, v. 3.1.2, and v. 4.1.0-exome) [141], CADD (v. 1.7) [138], dbSNP (v. 156 and previous versions) [135], AlphaMissense (v.04-2024) [142,143], and LiftOver [144] (online version at https://genome.ucsc.edu/cgi-bin/hgLiftOver, accessed on 20 September 2022).

### 4.5. Protein Folding

Protein models of NCSTN, PS1 (*PSEN1* gene), PEN-2 (*PSENEN* gene), and APH-1B (*APH1B* gene) were generated from the canonical Ensembl translated transcripts (i.e., ENST00000294785.10, ENST00000324501.10, ENST00000587708.7, and ENST00000261879.10, respectively) using the AlphaFold tool (v. 2.1.2) [145], which is freely available on the Galaxy France server (v. 22.01) (https://usegalaxy.fr/, accessed on 15 April 2022). The mutated models were processed in the same way, although we acknowledge the limitations of this tool for predicting the consequences of missense mutations [146,147]; it remains the best tool for predicting three-dimensional structures. The visualization and manipulation of the .pdb files was conducted via the RasTop tool (v.2.2) [148], and their annotation was a mix between InterPro (v.90.0) [149] predictions and cryo-EM structures of γ-secretase (models 2KR6 [150], 6IYC [151] & 5FN2, 5FN3, 5FN4, and 5FN5 [150]) available on the RCSB Protein Data Bank [152].

## Figures and Tables

**Table 1:** Characteristics of the 100 HS patients.

Individual Characteristics	Male	Female	All	*NA*
N	36	64	100	*-*
Age (mean ± sd)	34.9 ± 13.6	34.6 ± 11.1	34.7 ± 11.9	*1*
BMI (mean ± sd)	26.1 ± 4.5	27.1 ± 5.1	26.7 ± 4.9	*4*
Smoking status	21 (65.6%)	44 (71.0%)	65 (69.1%)	*6*
Familial case	7 (36.8%)	21 (37.5%)	28 (37.3%)	*25*
IBD history	0	1 (1.8%)	1 (1.3%)	*25*
Rheumatological history	0	2 (3.6%)	2 (2.7%)	*25*
**Hurley**				
I (mild)	4 (12.5%)	11 (17.2%)	15 (15.6%)	*4*
II (moderate)	12 (37.5%)	30 (46.9%)	42 (43.7%)	
III (severe)	16 (50.0%)	23 (35.9%)	39 (40.6%)	

BMI: Body Mass Index; sd: standard deviation; IBD: Inflammatory Bowel Disease; NA: Not Available data.

**Table 2:** All 91 γ-secretase variants found in the literature and the eight new variants identified in our cohort.

Gene	ID	Position (GRCh38)	Ex.	c/p.HGVS	Eff.	rsID	R.	Or.	F/S	Association
APH1A (ENST00000369109)	1	1:150267780	3	p.D98E	mis|spl	rs996158631	0	div.	–	–
APH1B (ENST00000261879)	**2°^N^**	**15:63277702**	**1**	**p.T27A**	**mis**	**rs77834210**	**0**	FR*	**S**	**–**
	**3°^N^**	**15:63279258**	**2**	**p.L71V**	**mis**	**–**	**0**	FR*	**S**	**–**
	4	15:63302375	5	p.H170R	mis	rs139355584	2	div.	F	–
	**5°^N^**	**15:63302416**	**5**	p.K185Sfs*10	**fs**	**rs745918508**	**0**	FR*	**S**	**–**
	6	15:63305770	6	p.R255C	mis	rs142676640	0	div.	–	–
NCSTN (ENST00000294785)	7	1:160343408	1	p.G6Vfs*22	fs	rs1266104510	0	div.	–	–
	8	1:160343434	1	p.G13Efs*15	fs	–	3	DE	S	AC
	**9°^N^**	**1:160343443**	**1**	p.L17Sfs*30	**fs**	**–**	**0**	FR*	**S**	**–**
	10	1:160344733	2	p.G33R	mis|spl	–	6	JP	F	–
	11	1:160344767	2	p.L44*	non	–	0	BR	F	DDD
	12	1:160344818	2	p.G61V	mis	–	3	FR*|MT*	F	–
	13	1:160349018	3	p.V72Yfs*16	fs	rs1243425689	10	CN	F	–
	14	1:160349031	3	p.V75I	mis	rs12045198	10	CN	F	–
	**15°**	**1:160349039**	**3**	**p.E77D**	**mis**	**rs35603924**	**0**	div.|FR*	**S**	**–**
	16	1:160349086	3	p.P93Lfs*15	fs	–	8	CN	FS	SAPHO
	17	1:160349578	4	p.T115Nfs*20	fs	–	9	FR*	–	PASH
	18	1:160349583	4	p.R117*	non	rs387906896	10	CN|US*|AfUS	F	–
	19	1:160349799	4-5	c.436+129A>G	int	rs2274184	0	SG	FS	–
	20	1:160350115	5	p.N150Ifs*52	fs	–	2	CN	F	–
	21	1:160350118	5	p.S151Qfs*48	fs	–	4	CN	F	–
	22	1:160350145	5	p.C159*	non	–	9	CN|GR*	F	–
	23	1:160350150	5	p.I162Yfs*40	fs	–	1	IT*	S	PASH/SAPHO
	24	1:160350155	5	p.Q163Sfs*39	fs	–	10	FR	F	–
	25	1:160350165	5	p.S166*	non	–	6	CN	F	–
	26	1:160350221	5	p.D185N	mis	rs201293070	9	GB*	S	Diab
	27	1:160350251	5-6	c.582+1del(G)	spl*	–	10	JP	F	–
	28	1:160350251	5-6	c.582+1G>A	spl	rs1373027391	0	SG	FS	–
	29	1:160351256	6	p.S206*	non	–	7	CN*	F	–
	30	1:160351271	6	p.P211R	mis	–	10	CN	S	–
	31	1:160351286	6	p.Q216P	mis	–	9	CN|MT*	F	–
	32	1:160351310	6	p.V224_T227del	del	–	3	MT	F	–
	33	1:160351325	6	p.C230Pfs*32	fs	–	8	CN|IN	F	AC
	34	1:160351713	7	p.L251Vfs*2	fs	–	3	CN	F	Com
	35	1:160351755	7	p.T265Nfs*8	fs	–	2	CN	F	–
	36	1:160352097	8	p.E296G	mis	rs758910156	7	CN|MT*	F	–
	**37°^N^**	**1:160352104**	**8**	p.A300Wfs*20	**fs**	**–**	**0**	FR*	**F**	**–**
	38	1:160352154	8	p.A315V	mis	rs1553210405	9	CN	F	–
	39	1:160352188	8	p.M326Ifs*31	fs	–	5	SG	–	–
	40	1:160352207	8-9	p.E333Q367del(x9)	spl*	–	5	GB	F	–
	41	1:160352213	8-9	c.996+7G>A	int	rs202046846	9	GB*|FR*	S	AC|Diab
	42	1:160352992	9-10	c.1101+1G>A	spl	rs1347055289	10	GB	F	–
	43	1:160353001	9-10	c.1101+10A>G	int	rs1048828525	9	GB*	S	–
	44	1:160353198	10	p.D381Sfs*7	fs	–	1	IT*	S	PAPASH
	**45°**	**1:160354113**	**10-11**	**c.1180-5C>G**	**int**	**rs7528638**	**5**	GB|FR*	**F**	**–**
	46	1:160354167	11	p.A410V	mis	rs147225198	6	US*	S	–
	**47°**	**1:160354187**	**11**	**p.N417Y**	**mis**	**rs143039637**	**0**	div.|FR*	**S**	**–**
	48	1:160354190	11	p.Q418*	non	–	0	div.	–	–
	49	1:160354196	11	p.Q420*	non	–	10	CN|SG|div.	F	–
	**50°**	**1:160354223**	**11**	p.R429*	**non**	**rs771414318**	**5**	JP|FR*	**F**	**–**
	51	1:160354232	11	p.R432*	non	–	3	CN*	FS	KTS
	52	1:160354238	11	p.R434*	non	rs1085307081	10	CN|ES*|FR	F	DDD
	53	1:160354263	11	p.D443Lfs*6	fs	–	3	ES*	–	DDD
	54	1:160354291	11-12	c.1352+1G>A	spl	–	9	CN	F	–
	55	1:160354194	11	c.1381del(C)	fs	–	2	FR*	F	–
	56	1:160355941	13	p.Q512*	non	–	3	CN*	F	–
	57	1:160355959	13-14	p.A486_T517del(x13)	spl*	rs1553210984	9	CN	F	–
	58	1:160356263	14	p.T519Nfs*10	fs	–	3	CN	S	–
	59	1:160356343	14	p.Y545*	non	–	8	IR	F	PASH
	60	1:160356655	15	p.Y565*	non	–	10	CN	F	–
	61	1:160356662	15	p.Q568*	non	–	9	JP	F	–
	62	1:160356687	15	p.G576V	mis	–	3	FR*|MT*	F	–
	63	1:160356707	15	p.R583*	non	–	3	IT*	F	DDD
	64	1:160356712	15	p.E584Dfs*44	fs	rs1553211087	10	CN	F	–
	65	1:160356728	15	p.S590G/p.S590Afs*3	mis|spl	–	10	FR	F	–
	66	1:160357046	16	p.Y600*	fs	–	6	CN|IN	FS	AC
	67	1:160357122	16	p.R626*	non	–	3	ES*|IN	F	DDD|AC
	68	1:160357161	16	p.Q639Gfs*31	fs	–	5	NL	F	–
	**69°^N^**	**1:160357189**	**16**	p.W648*	**non**	**–**	**0**	FR*	**S**	**–**
	70	1:160358788	3′	c.*517_*518del(CA)	utr	rs141849450	4	CN	S	–
PSEN1 (ENST00000324501)	71	14:73186897	6	p.S178Ffs*10	fs	rs1174374799	0	div.	–	–
	72	14:73192820	7	p.P242Lfs*11	fs	rs1595035030	10	CN	F	–
	**73°**	**14:73198145**	**8-9**	**c.868+16G>T**	**int**	**rs165932**	**4**	CN|div.|FR*	**S**	**Ps**
	**74°**	**14:73206470**	**9**	**p.E318G**	**mis|spl**	**rs17125721**	**8**	GB|div.|FR*	**F**	**–**
	**75°^N^**	**14:73211786**	**10**	**p.Q325E**	**mis**	**–**	**0**	FR*	**S**	**–**
	76	14:73217163	11	p.S390Efs*20	fs	–	3	FR*	F	Crohn
	**77°**	**14:73217252**	**11-12**	**c.1248+8T>C**	**int**	**rs362382**	**0**	div.|FR*	**S**	**–**
PSEN2 (ENST00000366783)	**78°^N^**	**1:226882007**	**4**	**p.G34S**	**mis**	**rs200636353**	**0**	FR*	**S**	**–**
	79	1:226894073	12	p.T380K	mis	rs143912759	0	div.	–	–
PSENEN (ENST00000587708)	80	19:35745943	2	p.R5*	non	–	3	CN	F	–
	81	19:35745965	2	p.L12*	non	rs1555738763	5	DE	–	DDD
	82	19:35745973	2	p.C15Pfs*101	fs	–	5	JP	F	AC
	83	19:35746418	2-3	p.G21_Y56del(x3)	spl*	rs1555738836	4	IN	F	DDD
	84	19:35746418	2-3	c.62-1G>T	spl	–	5	IN*	F	DDD
	85	19:35746423	3	p.F23Vfs*98	fs	rs1555738837	10	GB*|FR*	FS	DDD
	86	19:35746423	3	p.F23Lfs*46	fs	rs1555738837	10	CN	FS	DDD|Ps
	87	19:35746441	3	p.L29Sfs*92	fs	–	3	TH	F	DDD
	88	19:35746472	3	p.R39*	non	–	5	DE	–	DDD
	89	19:35746525	3-4	p.G21_Y56del(x3)	spl*	rs1970575677	5	FR	F	DDD
	90	19:35746706	3-4	c.167-2A>G	spl	rs1555738903	7	CN	F	DDD
	91	19:35746709	4	p.Y56*	non	rs751542345	8	Jash|FR*	F	DDD|IBD|PS
	92	19:35746735	4	p.L65R	mis	rs1555738906	7	CN	F	Com|DDD
	93	19:35746756	4	p.S73Pfs*72	fs	–	1	DE	–	DDD
	94	19:35746769	4	p.I77Hfs*45	fs	–	1	CN	F	PASH
	95	19:35746770	4	p.I77Tfs*45	fs	–	4	CN*	F	DDD
	96	19:35746812	4	p.Y91Tfs*54	fs	–	2	div.	F	–
	97	19:35746819	4	p.F94Sfs*51	fs	rs1555738943	9	CN	F	–
	98	19:35746833	4	p.L98Wfs*47	fs	–	2	div.	F	–
	99	19:35746845	4	p.*102Rxt*50	elg	–	3	FR*	–	DDD|Diab

This table concerns the following γ-secretase genes: *APH1A* [17], *APH1B* [16,17,36], *NCSTN* [12,17,24,36,41,42,53,54,55,56,57,58,59,60,61,62,63,64,65,66,67,68,69,70,71,72,73,74,75,76,77,78,79,80,81,82,83,84,85,86,87,88,89,90,91,92,93,94,95,96,97,98,99,100,101], *PSEN1* [17,36,54,55,59,73,102], *PSEN2* [17], and *PSENEN* [16,17,56,59,73,90,102,103,104,105,106,107,108,109,110,111,112,113]. The lines with an (°) and in **bold** correspond to the above-mentioned variants (15) found in our HS cohorts. Those with an (^N^) are the new ones (8), not mentioned in the literature. Ex.: exons; Eff.: effect (del, elg, fs, int, mis, non, spl, spl*, and utr meaning respectively deletion with no frameshift, elongation, frameshift, intronic variant, missense, nonsense, splice site, known alternative splicing event, and untranslated region variant); R.: number of studied reviews [13,59,77,87,99,101,114,115,116,117,118] (out of 11) citing this mutation; Or.: origin. The two-letter country code was used for the various studies (BR: Brazil; CN: China; DE: Germany; ES: Spain; FR: France; GB: United Kingdom; IN: India; IR: Iran; IT: Italy; JP: Japan; MT: Malta; NL: Netherlands; SG: Singapour; TH: Thailand; US: United States and AfUS and Jash for African-American and Jewish Ashkenazi populations). When the code is followed by an asterisk (*), it indicates that the population is not explicitly mentioned in the article, and the country is inferred based on the authors’ affiliations; F/S: familial and/or sporadic case. The last column lists the disease and/or syndrome associations mentioned in the articles—all acronyms are defined in Supplementary Figure S1.

**Table 3:** Sequencing protocols (Agilent Technologies, Inc., Santa Clara, CA, USA).

Cohort	Agilent WES Kit	Protocol	Coverage
HS1	SureSelectXT Human All Exon V4+UTR	2 × 100 bp	75×
HS2	SureSelectXT Human All Exon V6	2 × 150 bp	120×
CTL	SureSelectXT Human All Exon V5+UTR	2 × 100 bp	75×

## Data Availability

The datasets presented in this article are not readily available because the data are part of an ongoing study. Requests to access the datasets should be directed to Sophie Hüe or Kévin Muret.

## Supplementary Materials

The supporting information can be downloaded at: https://www.mdpi.com/article/10.3390/ijms251910374/s1. References [4,5,6,7,8,13,14,15,16,17,18,19,20,21,22,23,24,25,26,27,28,29,30,31,32,33,34,35,36,37,38,39,40,41,42,43,44,45,46,47,48,59,71,72,77,87,99,101,114,115,116,117,118,153,154,155,156] are cited in the supplementary materials.
