# Supplementary material for: Comprehensive Catalog of Variants Potentially Associated with Hidradenitis Suppurativa, Including Newly Identified Variants from a Cohort of 100 Patients
Source: Int J Mol Sci. 2024 Sep 26;25(19):10374. doi: 10.3390/ijms251910374 (PMC11476843; doi:10.3390/ijms251910374)
Supplement: Supplementary file 1 [file ijms-25-10374-s001.zip › ijms-3168929 Supplementary Fig & Tables.pdf]

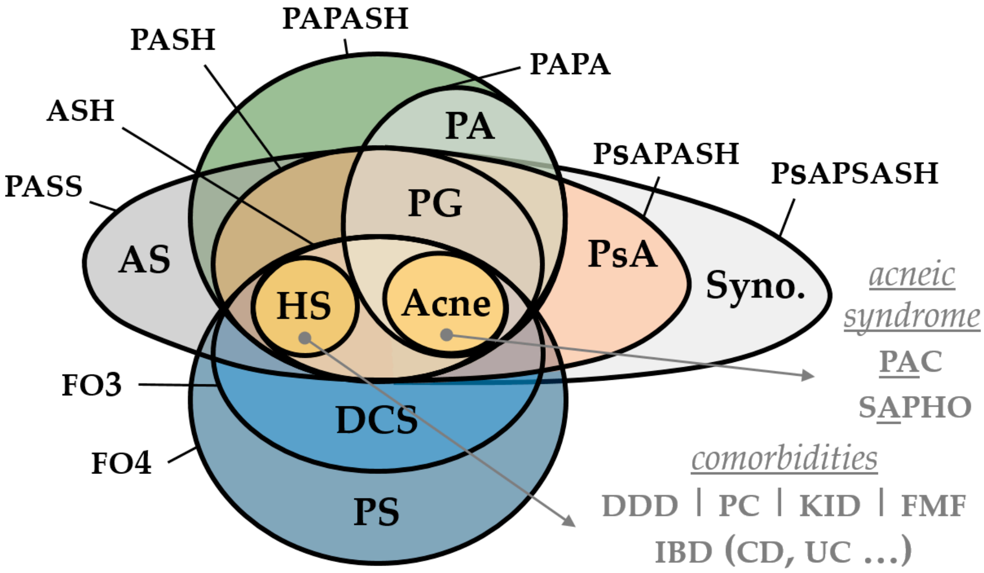

Supplementary Figure S1. Comorbidities of HS and its involvement in various autoinflammatory syndromes [4–8]. Diseases and syndromes: AS: ankylosing spondyloarthritis; ASH: acne, hidradenitis suppurativa; CD: Crohn's disease; DCS: dissecting cellulitis of the scalp; DDD: Dowling-Degos disease; FMF: familial Mediterranean fever; FO3: follicular occlusion triad (hidradenitis suppurativa, acne conglobata, dissecting cellulitis of the scalp); FO4: follicular occlusion tetrad (hidradenitis suppurativa, acne conglobata, dissecting cellulitis of the scalp and pilonidal sinus); HS: hidradenitis suppurativa; IBD: inflammatory bowel disease; KID: keratitis, ichtyosis, deafness; PA: pyogenic arthritis; PAC: pyoderma gangrenosum, acne, ulcerative colitis; PAPA: pyogenic arthritis, pyoderma gangrenosum, acne; PASH: pyoderma gangrenosum, acne, hidradenitis suppurativa; PAPASH: pyogenic arthritis, pyoderma gangrenosum, acne, hidradenitis suppurativa; PASS: pyoderma gangrenosum, acne, hidradenitis suppurativa, ankylosing spondyloarthritis; PC: pachyonychia congenita; PG: pyoderma gangrenosum; PS: pilonidal sinus; PsA: psoriatic arthritis; PsAPASH: psoriatic arthritis, pyoderma gangrenosum, acne, hidradenitis suppurativa; SAPHO: synovitis, acne, pustulosis, hyperostosis, osteitis; Syno.: synovitis; UC: ulcerative colitis.

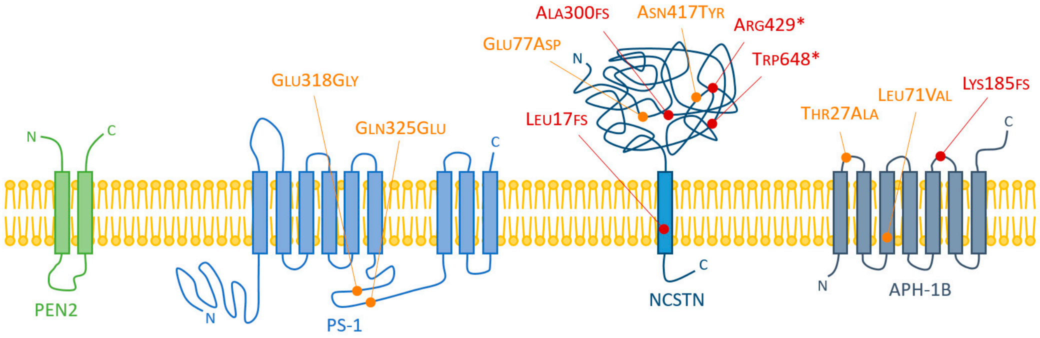

Supplementary Figure S2. All γ-secretase exonic variants identified in our HS1 & HS2 cohorts. In orange and red, variants with moderate and strong impacts. FS: frameshift variant; *: nonsense variant.

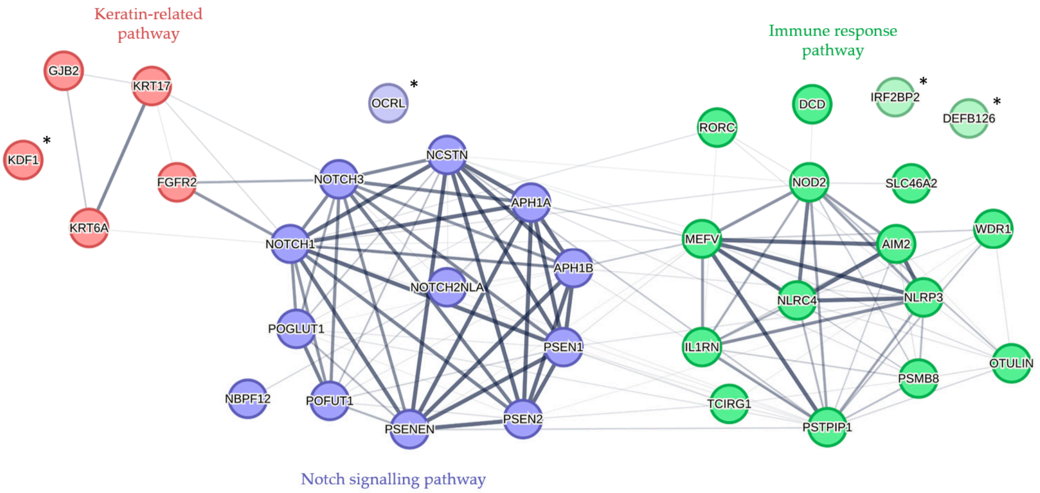

Supplementary Figure S3. Protein network of all genes discussed in this article, according to STRING (v. 12.0). Three clusters are identified: the Notch signalling pathway, the immune response pathway and keratinization (consistent with Jfri *et al.* [118]). The interaction score is set to 0.150 to enhance sensitivity over specificity. The thicker the edges, the stronger the connections within the clusters. (*) indicates genes not connected to the network but guilt-by-association [153–155].

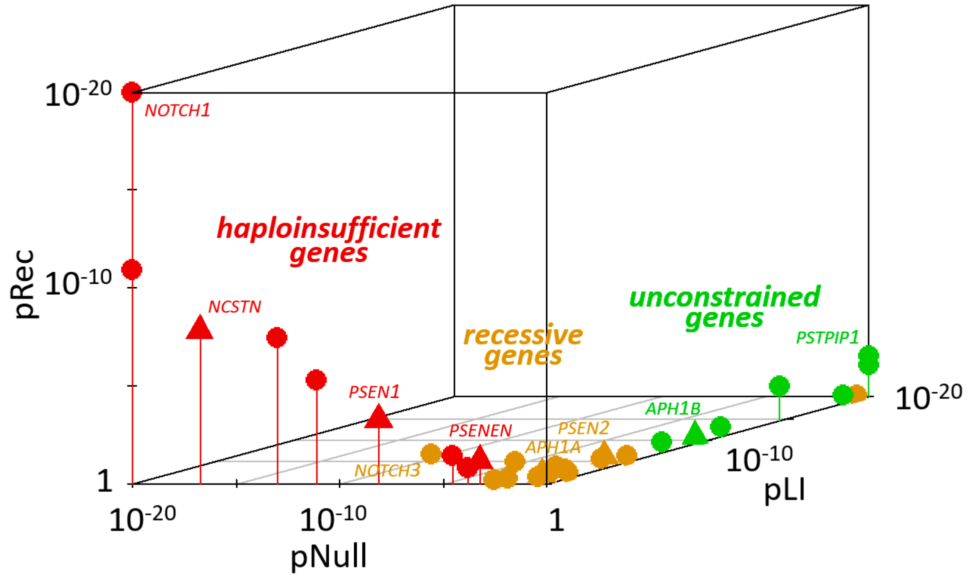

Supplementary Figure S4. Distribution of pLI (probability of intolerance to loss of function), pRec (probability of being recessive) and pNull (probability of being unconstrained) gene scores. Scores were obtained from the gnomAD (v4.1) database. Extreme values are capped to 10^{-20} to enhance the readability of the figure. The triangles (▲) and circles (●) correspond to the genes of the γ-secretase complex and other genes mentioned in this review, respectively. From left to right, the genes subject to haploinsufficiency (in red) are: *NOTCH1*, *FGFR2*, *NCSTN*, *SOX9*, *RORC*, *PSEN1*, *KLF5*, *PSENEN*, and *KDF1*.

Supplementary Table S2. Example of a mutation presented in 9 distinct articles.

Article	MANE Transcript	Exon	Genome	Position	c.HGVS	p.HGVS
[72]	**NM_001290184**	**4**	**-**	**-**	**c.218delC**	**p.P73Lfs*15**
[71]	**NM_015331**	**3**	**-**	**-**	**c.278delC**	**p.P93Lfs*15**
[116]	-	-	-	-	c.218delC	-
[78]	-	4	-	-	c.218delC	p.P73Lfs*15
[59]	-	-	-	-	c.218delC	p.I73Tfs*3
[115]	-	-	-	-	c.218delC	p.P73Lfs*15

[114]	-	4	-	-	c.218delC	p.P73Lfs*15
	-	-	-	-	c.278delC	p.P93Lfs*15
[13]	-	3	-	-	c.218delC	p.P93Lfs*15
	-	3	-	-	c.278delC	p.P93Lfs*15
[101]	-	-	-	-	c.218delC	p.P73Lfs*15
	-	-	-	-	c.278delC	p.P93Lfs*15
Current article	*ENST000000294785 (= NM_015331)*	*3*	*GRCh38*	*1:160349086*	*c.278delC*	*p.P93Lfs*15*

The first two lines in **bold** represent the two original articles from 2018 reporting first this mutation. The dashes indicate missing data that would have been helpful for tracking the mutation over the past five years. This example illustrate a deletion in *NCSTN* described simultaneously in 2018 in two independent articles and in different patients. The annotations differ: the first laboratory reports a c.218delC mutation (p.P73Lfs*15) on the NM_001290184 transcript affecting exon 4 [35] while the second laboratory reports a c.278delC mutation (p.P93Lfs*15) on the NM_015331 transcript affecting exon 3 [34]. Although both annotations are technically correct, they initially appear to describe distinct mutations. However, subsequent reviews have removed the transcript details, altered the protein HGVS code (p.P73Lfs*15 → p.I73Tfs*3 → p.P93Lfs*15), introduced new errors regarding the exon (4 → 3), and included the mutations separately, despite them being the same mutation. This highlights the importance of clarifying such details, always specifying the reference transcript (preferably using the standard MANE transcript) or at least the genome version and variant position. Errors are common in such catalogs, and we hope this revised version will facilitate future research on HS.

Supplementary Table S3. Additional polymorphisms in other genes associated with HS (78) in the literature, including the two new variants identified in our cohort

Gene	ID	Position (GRCh38)	Ex.	c/p.HGVS	Eff.	rsID	R.	Or.	F/S	Association
AIM2 (ENST00000368130)	100	1:159076820	5'UTR	-208A>C	–	rs41264459	0	IT	S	(PA)PASH❘SAPHO
DCD (ENST00000293371)	101	12:54645237	4	p.A76Sfs*21	fs	rs538180888	0	IT	F	–
DEFB126 (ENST00000382398)	102	20:145459	2	p.K35*	non	rs142956939	0	div.	F	–
FGFR2	103	10:121565644	3	p.S57L	mis	rs56226109	0	div.	–	–
(ENST00000358487)	104	10:121551422	5	p.K164N	mis	–	2	GB*	F	AC❘Com
GJB2	105	13:20189548	2	p.G12R	mis	rs104894408	0	US*❘IT*	S	KID❘FO3
(ENST00000382848)	**106°**	**13:20189552**	**2**	**p.G12Vfs*2**	**fs**	**rs80338939**	**1**	**IT*❘FR***	**S**	**PASH**
	**107°**	**13:20189503**	**2**	**p.V27I**	**mis**	**rs2274084**	**0**	**JP-AfUS❘FR***	**S**	**KID❘FO3**
	108	13:20189463	2	p.A40V	mis	–	1	US*❘IT*	S	KID❘FO3
	109	13:20189434	2	p.D50N	mis	rs28931594	0	AfUS❘IT*	–	KID❘FO3
	110	13:20189434	2	p.D50Y	mis	rs28931594	0	IT*	S	KID❘FO3
	**111°**	**13:20189241**	**2**	**p.E114G**	**mis**	**rs2274083**	**0**	**JP-AfUS❘FR***	**S**	**KID❘FO3**
IL1RN (ENST00000409930)	**112°**	**2:113132707**	**4**	**p.A124T**	**mis**	**rs45507693**	**1**	**IT*❘FR***	**S**	**PAPASH**
IRF2BP2 (ENST00000366609)	113	1:234608830-234608870	1	p.A209Qfs*31	fs	–	0	FI*	F	CVID
KDF1 (ENST00000320567)	114	1:26951628	2	p.F251L	mis	rs1057519508	0	SA	F	Ectodermal Dysplasia
	115	1:26951621	2	p.H254Y	mis	–	0	FR*	S	Ectodermal Dysplasia
~KLF5 / LINC00393 (ENST00000443621)	116	13:73432270	– / 1-2	g.73432270A>G	– / int	rs17090189	0	US*	FS	diverse
KRT6A (ENST00000330722)	117	12:52488362	7	p.T464P	mis	rs61293647	1	ES*	S	PC
KRT17	118	17:41624235	1	p.N92S	mis	rs59151893	0	IT*	F	FO4❘PC
(ENST00000311208)	119	17:41624226	1	p.L95P	mis	rs28928899	0	CN*	S	FO4❘PC❘AS

MEFV (ENST00000219596)	120	16:3254338	2	p.E244K	mis	rs1959081392	0	ES	S	PAAND
	**121°**	**16:3254626**	**2**	**p.E148Q**	**mis**	**rs3743930**	**0**	**TR|FR***	**FS**	**FMF|Pilonidal sinus**
	122	16:3249675	3	p.S339F	mis	rs104895157	0	TR	S	–
	**123°**	**16:3249586**	**3**	**p.P369S**	**mis**	**rs11466023**	**0**	**TR|FR***	**S**	**Robinow**
	**124°**	**16:3249468**	**3**	**p.R408Q**	**mis**	**rs11466024**	**0**	**TR|FR***	**S**	**Robinow**
	**125°**	**16:3243880**	**9**	**p.I591T**	**mis**	**rs11466045**	**0**	**IT*|FR***	**S**	**PASH**
	126	16:3243447	10	p.M680I	mis	rs28940580	1	TR	FS	PS|SAPHO
	127	16:3243407	10	p.M694V	mis	rs61752717	1	MD|TR	FS	PAPASH|FMF|FO3
	128	16:3243403	10	p.K695R	mis	rs104895094	0	div.	–	–
	129	16:3243310	10	p.V726A	mis	rs28940579	4	AM|MD|TR	FS	PAPASH|FO3|FMF
NBPF12 (ENST00000698835)	130	1:146960267	7	p.C42S	mis	rs1345358545	0	div.	F	–
NF1P6 (ENST00000426025)	131	22:15627718	4-5	n.602-2C>A	spl	rs776018604	0	div.	F	–
NLRC4 (ENST00000402280)	132	2:32251323	4	p.R181*	non	rs759551435	1	IT*	S	PAPASH
	133	2:32235515	8	p.C890R	mis	rs544969923	1	IT*	S	PASH/SAPHO
NLRP3 (ENST00000336119)	**134°**	**1:247425556**	**4**	**p.Q703K**	**mis**	**rs35829419**	**1**	**IT*|FR***	**S**	**PASH**
NOD2 (ENST00000647318)	135	16:50699832	2	p.A113T	mis	rs34684955	0	div.	–	–
	136	16:50710966	4	p.H325R	mis	rs5743272	0	div.	–	–
	137	16:50711532	4	p.R514W	mis	rs576658764	0	div.	–	–
	**138°**	**16:50712015**	**4**	**p.R675W**	**mis**	**rs2066844**	**2**	**IT*|FR***	**S**	**PASH**
	139	16:50712034	4	p.R681H	mis	rs35285618	0	div.	–	–
	**140°**	**16:50712085**	**4**	**p.A698G**	**mis**	**rs5743278**	**0**	**FR***	**S**	**–**
	**141°**	**16:50712280**	**4**	**p.R763Q**	**mis**	**rs5743279**	**0**	**FR***	**S**	**–**
	142	16:50722629	8	p.G881R	mis|spl	rs2066845	3	IT*	S	PASH
	**143°**	**16:50722660**	**8**	**p.A891D**	**mis**	**rs104895452**	**0**	**FR***	**S**	**–**
	144	16:50725529	10	p.L948V	mis	rs1337759230	1	AM	F	FO3
	**145°**	**16:50729868**	**11**	**p.L980Pfs*2**	**fs**	**rs2066847**	**1**	**IT*|FR***	**S**	**PASH**
NOTCH1 (ENST00000651671)	146	9:136510652	17-18	c.2740+1G>T	spl	–	0	AfUS	–	Keratoacanthoma
NOTCH3 (ENST00000263388)	**147°N**	**19:15197571**	**2**	**p.C43Lfs*32**	**fs**	**rs749829137**	**0**	**FR***	**S**	**–**
	148	19:15192130	4	p.H170R	mis	rs147373451	0	div.	F	–
	149	19:15186898	12	p.V644D	mis	rs148046938	0	div.	F	–
NOTCH2NLA (ENST00000362074)	150	1:146189383	1-2	c.-44-2A>G	spl	rs3872062	0	div.	F	–
OCRL (ENST00000371113)	151	X:129562396	11	p.R318C	mis	rs137853263	1	IT*	F	DD2
	152	X:129569274	15	p.R493W	mis	rs137853846	1	IT*	–	DD2
	153	X:129569364	15	p.D523N	mis	–	1	IT*	F	DD2
OTULIN (ENST00000284274)	154	5:14673698	2	p.I70T	mis	rs745829522	1	IT*	S	PASH
	**155°**	**5:14681484**	**4**	**p.Q115H**	**mis**	**rs147790160**	**1**	**IT*|FR***	**S**	**PASH**
POFUT1 (ENST00000375749)	156	20:32216608	3-4	c.430-1G>A	spl	rs958172940	4	ES*	S	DDD
	157	20:32230974	6	p.W297*	non	–	1	ES*	F	DDD
POGLUT1 (ENST00000295588)	158	3:119490567	9	p.R272*	non	rs747897279	1	FR*	F	DDD
PSTPIP1 (ENST00000558012)	159	15:76995150	5'	c.-421CCTG[6] [5to6-rep]	µsat	rs55909412	0	DE	S	PASH
	160	15:76995150	5'	c.-421CCTG[8] [5to8-rep]	µsat	rs55909412	3	TR|RU|FR*	FS	(PA)PASH, PCAS
	161	15:77032304	11	p.E250Q	mis	rs28939089	1	DE*	F	PAPASH
	162	15:77032320	11	p.T255M	mis	rs766895096	1	FI*	–	Diab|PG|UC
	**163°**	**15:77032329**	**11**	**p.G258A**	**mis**	**rs34240327**	**0**	**div.|FR***	**S**	**–**
	164	15:77032387	11	p.E277D	mis	rs990986006	5	MD	S	PAPASH/FMF
	165	15:77032439	12-13	c.838+45C>[AGT]	int	rs116895455	0	SG	FS	–
	166	15:77035850	14	p.Y345C	mis	rs1192521928	3	JP	F	PASH
	**167°**	**15:77035928**	**14**	**p.T371I**	**mis|spl**	**rs34908107**	**0**	**div.|FR***	**FS**	**diverse**
	168	15:77037018	14-15	c.1120-27G>[AC]	int	rs766351379	0	SG	FS	–
	**169°**	**15:77037069**	**15**	**p.A382T**	**mis**	**rs145344175**	**0**	**div.|FR***	**FS**	**diverse**
	170	15:77037133	15	p.G403E	mis	rs201572812	0	div.	FS	diverse
	171	15:77037138	15	p.R405C	mis	rs201253322	4	div.|ES*	FS	PASH
	172	15:77037146	15	p.F407L	mis	rs200363654	0	SG	FS	–
PSMB8 (ENST00000374882)	**173°**	**6:32843975**	**1**	**p.G8R**	**mis**	**rs114772012**	**0**	**IT*|FR***	**S**	**AS|PASH|SAPHO**

RORC (ENST00000318247)	**174°**	**1:151831737**	**1**	**p.R10***	**non**	**rs17582155**	**0**	**div.∣FR***	**F**	**–**
SLC46A2	175	9:112890266	1	p.A139Gfs*39	fs	–	0	div.	F	–
(ENST00000374228)	**176°[N]**	**9:112889705**	**1**	**p.A326Gfs*133**	**fs**	**rs1841700210**	**0**	**FR***	**S**	**–**
~SOX9	177	17:71515958	–	g.71515958G>A	–	rs10512572	0	US*	FS	diverse
TCIRG1 (ENST00000265686)	178	11:68044207	9	p.Q295*	non	–	0	div.	F	–
WDR1 (ENST00000499869)	**179°**	**4:10087915**	**8**	**p.H248R**	**mis**	**rs41268387**	**1**	**IT*∣FR***	**S**	**PASH/SAPHO**

List of genes outside the γ-secretase complex: *AIM2* [14], *DCD* [15], *DEFB126* [16], *FGFR2* [17,18], *GJB2* [19–24], *IL1RN* [25], *IRF2BP2* [26], *KDF1* [27,28], *KRT6A* [29], *KRT17* [30,31], *MEFV* [17,24,25,32–34], *NBPF12* [16], *NF1P6* [16], *NLRC4* [24], *NLRP3* [25], *NOD2* [17,24,25,33], *NOTCH1* [35], *NOTCH3* [16,36], *NOTCH2NLA* [16], *OCRL* [37], *OTULIN* [24], *POFUT1* [38,39], *POGLUT1* [40], *PSTPIP1* [17,24,25,32,36,41–48], *PSMB8* [25], *RORC* [16], *SLC46A2* [16], *TCIRG1* [16], and *WDR1* [24]. Two variants were detected near *KLF5* and *SOX9* using GWAS approaches [156]. The lines with an (°) and in **bold** correspond to the above-mentioned variants (23) found in our HS cohorts. Those with an ([N]) are the new ones (2), not mentionned in the literature. Ex.: exons; Eff.: effect (µsat, fs, int, mis, non, and spl meaning respectively microsatellite, frameshift, intronic variant, missense, nonsense, and splice site variant); R.: number of studied reviews [13,59,77,87,99,101,114–118] (out of 11) citing this mutation; Or.: origin. The two-letter country code was used for the various studies (AM:Armenia; CN:China; DE:Germany; ES:Spain; FR:France; GB:United Kingdom; IT:Italy; JP:Japan; MD:Moldova; RU:Russia; SA:Saudi Arabia; SG:Singapour; TR:Turkey; US:United States and AfUS for African-American populations). When the code is followed by an asterisk (*), it indicates that the population is not explicitly mentioned in the article, and the country is inferred based on the authors' affiliations ; F/S: familial and/or sporadic case. The last column lists the disease and/or syndrome associations mentioned in the articles — all acronyms are defined in Supplementary Figure S1.
